# Special Building Materials

**Published:** 1919-08-23

**Authors:** 


### HOSPITAL CONSTRUCTION: THE NEW ERA.

### X. Special Building Materials.

The whole building world is stirred at the present moment with the problem of substitution—meaning by that not merely the search for temporary expedients to take the place of normal materials, but a much wider and worthier enquiry. Our old friends bricks and timber, tiles and stone have so greatly advanced in scarcity and price that it is legitimate, not to say urgently necessary, to discuss whether they cannot be supplanted by other devices. In buildings of a domestic, monumental, or academic character, there is a certain shrinking from the use of the unusual, a certain fear that novelty in material means slightly bad form; there need be no hesitations of this kind in the sphere of hospital architecture.

The construction of hospitals goes ever hand in hand with that progressive art—the art of healing —and need never fear that mere innovation, if it is based on a good cause, is a fault. The hospital architect, far from dreading progress, must welcome it to a degree beyond that attainable by the architect of certain other classes of work. It is, therefore, specially open to him to study, search out, test, and examine whatever comes within his knowledge as a means to this worthy end. This is no hit at "other" architecture; the writer happens to be engaged in both kinds, but he realises that in the domain of hospital design, there is a very special opening for the possibilities of drastic changes. It is for this reason that he has during recent months devoted some time to the special study of certain experiments which, if they cannot yet be called developments, are at least healthy strivings after a revolution in accepted methods.

The simplest aspect of the case is perhaps the idea, no longer new, of replacing ordinary brick and stone by ordinary ferro-concrete.

Now it may be admitted at once that if ferro-concrete can win a conspicuous victory over its older rivals in the matter of price, it is deserving of full consideration. It happens, however, that at the present time, and in the present state of prices, a conspicuous victory can only be won when by the scarcity or remoteness of bricks, plus the prevalence of good gravel almost on the site, concrete is put into a very favourable condition. If a builder has the luck to find good ballast-gravel in his excavation soil, so that he has neither to cart away rubbish earth nor to pay the transport of important aggregate, thus, unless bricks are very near at hand and very cheap, he may (with a reasonably priced cement) show such a balance in favour of concrete as to give that material, even when adequately reinforced, a lead over brickwork.

But unless that lead is substantial, the balance may still dip in favour of the older-fashioned methods, specially for two reasons. Concrete, particularly reinforced concrete, does not lend itself at all favourably to alteration when once erected. It may certainly be argued that a good architect should be able, with reasonable forethought, to design his building and delineate his design in such a way as to render alterations during progress or after completion unnecessary. This is true in a way, but hospital architecture is a progresive art, as we have pointed out, ever advancing with the new needs and new discoveries of medical science, and it may well happen that even during the erection of a building, new medical facts may come to light which make some modification of a design desirable. Again, hospitals often want enlarging, and sometimes it becomes necessary to devote to a new purpose a room designed for other uses. Thus, the difficulty of alteration becomes a very substantial drawback. Ferro-concrete cannot be hacked, cut into, or removed with the ease that brickwork permits. Further, concrete is a terrible friend to condensation. We believe that the experts who have been for many months experimenting almost feverishly in the interests of the cottage housing problem, have almost barred concrete on this very ground. It is a singularly unhomely material and we are not sure, though this is approaching a region of speculation, whether its very absence of porosity is not in some degree unfavourable to health. A well-known specialist upon rheumatic complaints once told the writer that there was no better opportunity of contracting rheumatism than that offered by living in a ferro-concrete house before it was properly dry.

This article, however, is not meant as a total condemnation of ferro-concrete methods. It is intended rather as the expression of a hope that experiments which are now being conducted may lead at length to some means of arriving at a definitely new departure in wall, floor, and roof construction.

Imagine a building in which the principle of construction is complete rigidity coupled with so entire a cohesion between horizontal and vertical parts (*i.e.* between floors, roof, and walls) that the ordinary dependence upon bulk for stability is set aside. In fact, conceive of a structure so arranged that the necessary gravity is obtained, *not* by the thickness of *parts*, but by the unity of the *whole*. This is a result already very nearly obtainable by modern methods of refined reinforcement. Add to this some device by which the resultant thinness of walls and roof is made watertight, windtight, and temperature-proof, and complete the good work by some simple overcoming of the condensation trouble. When these things are done—and the experimenters are hard at work—we may witness the production of a building method which, in spite of its intractibility for alteration purposes, may have so great a claim on our financial consciences as to oust the older methods for hospital purposes. Meanwhile, good luck to the experimenters.

**Previous articles appeared on Jan. 25, Feb. 8, March 1, 15, 29, April 19, May 17, June 14, & July 19, p. 411.**

